# A Ministry of Health, and Other Addresses

**Published:** 1880-01

**Authors:** 


A MINISTRY OF HEALTH and other Addresses, by Benjamin Ward Richardson, M.D., F.R.S., M.A., LL.D., F.S.A., Fellow of the Royal College of Physicians, etc. D. Appleton & Co., N. Y. 1879.

This is a series of nine addresses, in a neatly bound volume of three hundred pages, discussing medical, medico-legal and hygienic subjects. The order of the subjects is as follows: 1. A Ministry of Health. II. William Harvey. III. A Homily Clerico-Medical. IV. Learning and Health. V. Vitality, Individual and National. VI. The World of Physic. VII. Burial, Embalming and Cremation. VIII. Registration of Disease. IX. Ether drinking and Extra-Alcoholic Intoxication.

